# Simple compared to covariate-constrained randomization methods in balancing baseline characteristics: a case study of randomly allocating 72 hemodialysis centers in a cluster trial

**DOI:** 10.1186/s13063-021-05590-1

**Published:** 2021-09-15

**Authors:** Ahmed A. Al-Jaishi, Stephanie N. Dixon, Eric McArthur, P. J. Devereaux, Lehana Thabane, Amit X. Garg

**Affiliations:** 1 Lawson Health Research Institute, London, Ontario Canada; 2 Department of Health Research Methods, Evidence, and Impact, McMaster University, Hamilton, ON Canada; 3 ICES, London, Ontario Canada; 4 Department Medicine, Epidemiology and Biostatistics, Western University, London, ON Canada; 5 Department of Mathematics and Statistics, University of Guelph, Guelph, ON Canada

**Keywords:** Cluster randomized trial, Covariate-constrained, Randomization, Balanced allocation, Restricted randomization

## Abstract

**Background and aim:**

Some parallel-group cluster-randomized trials use covariate-constrained rather than simple randomization. This is done to increase the chance of balancing the groups on cluster- and patient-level baseline characteristics. This study assessed how well two covariate-constrained randomization methods balanced baseline characteristics compared with simple randomization.

**Methods:**

We conducted a mock 3-year cluster-randomized trial, with no active intervention, that started April 1, 2014, and ended March 31, 2017. We included a total of 11,832 patients from 72 hemodialysis centers (clusters) in Ontario, Canada. We randomly allocated the 72 clusters into two groups in a 1:1 ratio on a single date using individual- and cluster-level data available until April 1, 2013. Initially, we generated 1000 allocation schemes using simple randomization. Then, as an alternative, we performed covariate-constrained randomization based on historical data from these centers. In one analysis, we restricted on a set of 11 individual-level prognostic variables; in the other, we restricted on principal components generated using 29 baseline historical variables.

We created 300,000 different allocations for the covariate-constrained randomizations, and we restricted our analysis to the 30,000 best allocations based on the smallest sum of the penalized standardized differences. We then randomly sampled 1000 schemes from the 30,000 best allocations. We summarized our results with each randomization approach as the median (25th and 75th percentile) number of balanced baseline characteristics. There were 156 baseline characteristics, and a variable was balanced when the between-group standardized difference was ≤ 10%.

**Results:**

The three randomization techniques had at least 125 of 156 balanced baseline characteristics in 90% of sampled allocations. The median number of balanced baseline characteristics using simple randomization was 147 (142, 150). The corresponding value for covariate-constrained randomization using 11 prognostic characteristics was 149 (146, 151), while for principal components, the value was 150 (147, 151).

**Conclusion:**

In this setting with 72 clusters, constraining the randomization using historical information achieved better balance on baseline characteristics compared with simple randomization; however, the magnitude of benefit was modest.

**Supplementary Information:**

The online version contains supplementary material available at 10.1186/s13063-021-05590-1.

## Introduction

The cluster-randomized trial (CRT) study design is useful when the interventions are naturally implemented on groups of individuals [1, 2]. In contrast to individually randomized trials, CRTs randomly allocate groups rather than independent individuals. Simple randomization is the most basic and straightforward type of random allocation. Each “randomized unit” is assigned purely by chance. However, suppose the total number of randomized units is small (e.g., fewer than 20 units). In that case, simple randomization may result in a moderate to a high probability of imbalance between the trial arms [3]. In two-group, parallel-arm, individual-level trials, some have suggested that including at least 1000 participants per group is required to provide sufficient protection against the imbalance of baseline characteristics [4]. In the CRT setting, it is often impossible to have such a large number of randomized units. In a systematic review of 300 CRTs, 50% of trials randomly allocated fewer than 21 clusters, and 75% allocated fewer than 52 clusters [5].

Observing between-group differences in a trial’s baseline characteristics complicates the interpretation of observed treatment effects and threatens the trial’s internal validity [6–8]. Other randomization techniques may help minimize the risk of imbalance on baseline measured characteristics when using parallel arm CRT designs [8]. These techniques are described as “restricted” or “constrained” and include stratification, matching, minimization, and covariate-constrained randomization. All restricted methods require a priori knowledge about participating clusters and the baseline measures used for the restriction process.

Covariate-constrained randomization can provide a better baseline balance than other allocation methods (e.g., simple random allocation, stratification, and minimization), especially when the number of randomized units is small (e.g., less than 20 clusters) [3, 8–10]. This manuscript focuses on covariate-constrained randomization, where we constrained the randomization process using two sets of baseline characteristics (either constraining on a set of prognostic variables or principal components). Principal components are a small set of artificial variables that explain most of the variance about a larger group of variables.

Covariate-constrained randomization limits the potential schemes available for selection among all possible allocations (called the randomization space). This method simultaneously balances several measured cluster- or individual-level characteristics to ensure that the two treatment arms are similar at baseline [8, 9]. Briefly, the covariate-constrained randomization process includes (i) a priori identifying and specifying a limited number of key prognostic cluster- or individual-level variables associated with the outcome that will be used to constrain the randomization process (or a function of baseline characteristics, for example, principal components); (ii) when there are 20 or more clusters [7], either enumerating all or generating at least 100,000 allocation schemes; (iii) for each allocation scheme, estimating balance on the selected baseline characteristics according to some predefined balance metric (e.g., absolute differences, standardized differences, or another measure [11]); (iv) choosing a constrained randomization space containing a subset of allocations that are balanced on the constrained baseline characteristics (e.g., 10% of the best allocations [11–13]); and (v) randomly selecting one allocation scheme from the constrained randomization space that will be used for the trial.

There is a trade-off between the potential for a better balance achieved on the constrained baseline characteristics and the potential concerns with highly restricted randomization [9, 12]. These trade-offs can include (i) jeopardizing the appearance of impartiality, for example, if pairs of clusters always (or never) appear in the same arm [9, 12]; (ii) a departure from the nominal type I error when clusters with correlated outcomes have a very high or very low probability of being included in the same trial arm [9, 12]; and (iii) a loss in statistical power when variables used in the constrained randomization do not associate with the trial outcome [9, 12]. Also, covariate-constrained randomization uses historical data on recruited clusters to capture baseline information on demographics, patients’ medical histories, and historical rates of the outcomes [14–16]. However, historical data may represent a “population for randomization” that is different from the “trial population”; the data may be several months to years old at the time of randomization. In an “open cohort” setting, information available at the randomization date cannot account for new participants entering the cohort during the trial period. Thus, the balance achieved at randomization with historical information does not guarantee a balance of the baseline characteristics during the trial period. It is important to note that the randomization design (i.e., constrained variables) needs to be considered at the analysis stage [17–19].

We conducted this study to understand the best practices for randomizing hemodialysis centers into two parallel groups in Ontario, Canada. The lessons learned from this study will help our group make informed decisions about randomization processes for several CRTs that we plan to advance.

### Motivating example

The CRT is an attractive design in the hemodialysis setting, especially when implementing interventions at the dialysis center level [15, 20, 21]. In addition, the CRT design offers logistical and administrative advantages such as simplifying the trial organization when evaluating policy- or cluster-level intervention [1, 22].

Suppose that we wish to undertake a CRT with hemodialysis centers in Ontario, Canada. In this example, we used historical data from administrative data sources to conduct covariate-constrained randomization. The trial period was three years, from April 1, 2014, to March 31, 2017, with no active treatment. The primary outcome was a composite of time-to-first event for cardiovascular-related death or non-fatal major cardiovascular-related hospitalization (hospital admission for myocardial infarction, stroke, or congestive heart failure).

### Objectives

This paper compared randomization methods for a two-arm, parallel-group CRT with the intent that all individuals within a given randomized center receive the same treatment. We randomized a moderate number of clusters (i.e., hemodialysis centers) using either simple randomization or covariate-constrained randomization with pre-trial historical records (the population for randomization). We performed the randomization on a single date and allowed patients to enter the cohort throughout the study period. We compared simple randomization to covariate-constrained randomization on balance achieved on a set of baseline characteristics during a 3-year trial period (the trial population). We constrained either on prognostic variables or principal components.

Our secondary aim was to assess whether, in the absence of any intervention, the allocation schemes selected through the constrained randomization process preserved (i) a null treatment effect on the primary outcome and (ii) a 5% nominal type I error rate.

## Methods

### Design and setting

We used a CRT design of outpatient hemodialysis centers in Ontario, Canada, that cared for a minimum of 15 patients. In 2013, Ontario had approximately 13.5 million residents with universal healthcare and physician services [23]. In the same period, Ontario had 26 regional dialysis programs that oversaw over 100 hemodialysis centers caring for about 8000 in-center patients in the outpatient setting [24].

### Data sources

We ascertained center- and patient-level characteristics using records from linked healthcare databases in Ontario, Canada (Additional file 1: Appendix 1) [25–38]. These datasets were linked using unique encoded identifiers and analyzed at ICES [39].

### Patients

We included two populations of patients, the population for randomization and the trial population. The population for randomization included patients who were actively receiving in-center hemodialysis on April 1, 2013. The trial population included an open cohort of patients who received in-center hemodialysis on April 1, 2014, or began receiving in-center hemodialysis during the trial period.

### Baseline characteristics

We identified two cluster- and 86 individual-level (total 88) baseline characteristics to describe each cohort (Additional file 1: Appendix 2); the cluster-level characteristics included the center size and historical rate for the primary outcome. There were 23 continuous, 58 binary, and 14 categorical baseline characteristics. Nine continuous baseline characteristics were also featured as categorical variables. We created a new binary (or “dummy”) variable to indicate each level of a category’s presence or absence. In total, we evaluated 156 continuous or binary candidate baseline characteristics.

### Randomization process

#### Sequence generation

We randomly allocated the 72 hemodialysis centers into two groups in a 1:1 ratio on a single date. Initially, we generated 1000 random allocation schemes using simple (unconstrained) randomization that required no information on baseline characteristics. This number of random allocations produced an estimate within 0.5% accuracy of the true hazard ratio of 1.00 with a 5% significance level and a standard deviation of 0.08; note, the true hazard ratio is 1.00 because there is no active intervention [40]. Then, as an alternative, we performed the covariate-constrained randomization using pre-trial historical records, which ended April 1, 2013 (see details below). Using PROC PLAN in SAS version 9.4 (SAS Institute Inc., NC, Cary), we generated 300,000 unique allocation schemes of the 72 centers (Additional file 1: Appendix 3). Greene (2017) suggested performing at least 100,000 allocations when there are at least 20 clusters; with our computational capacity, we enumerated 300,000 allocations.

#### Covariate-constrained randomization

We performed the covariate-constrained randomization in the following series of steps using baseline characteristics of the population for randomization [6, 8, 9, 41].

- **Step 1**: Randomly selected 300,000 allocation schemes from the 4.43 x 10^20^ possible allocation schemes.
- **Step 2**: For each of the 300,000 allocation schemes, we restricted the randomization space using one of two constraining criteria [8].

i. We constrained the allocation on a set of 11 baseline characteristics deemed prognostic for the outcome, based on prior literature or clinical experience (Additional file 1: Appendix 4a).
ii. We constrained the allocation on principal components. A principal component analysis is a dimensionality reduction technique whereby a dataset with many variables is transformed into a smaller set of artificial variables (called principal components). These principal components ideally retain some or most of the meaningful properties of the original set of variables. We used the principal components to account for some of the variation in the observed data and as criterion variables in our constrained randomization process (Additional file 1: Appendix 4b).

We compared baseline differences between the two arms using standardized differences [42, 43], which describes the differences between group means or proportions relative to the pooled standard deviation.

- **Step 3**: For each allocation scheme from the population for randomization, we counted the number of constrained variables with a standardized difference greater than 10% and calculated the sum of the constrained variables’ standardized differences [42, 44]. We added a penalty of ten units to the sum of standardized differences for each imbalanced constrained variable. We imposed this penalty to favor allocation schemes that had the least number of imbalanced constrained baseline characteristics. For example, if the sum of standardized differences was two and three constrained variables were imbalanced, the penalized sum of standardized differences would be 32.

From the 300,000 randomization schemes, we constrained the randomization space to the 30,000 best allocation schemes, based on the smallest sum of the penalized standardized differences [11–13]. From the 30,000 best allocations, we randomly sampled 1000 allocations to reduce the computational time for analysis [11, 12].

### Statistical analysis

For the 1000 sampled schemes, we (i) estimated the percentage of times each center was allocated to each arm, (ii) estimated the percentage of times each combination of center pairs appeared in the same group [41], and (iii) calculated the standardized difference of all 156 baseline characteristics for the trial population. We then estimated the percentage of time each of the 156 baseline characteristics was balanced among the 1000 sampled randomization schemes, (iv) calculated the median (25th and 75th percentiles) number of baseline characteristics balanced for the trial population, and finally (v) estimated the unadjusted and adjusted hazard ratio between the randomized arms, for the time-to-first event of the composite outcome of cardiovascular-related death or a non-fatal cardiovascular-related hospitalization (see definition of outcome in Additional file 1: Appendix 5; this is a primary outcome for future trials that is highly relevant to patients and their providers). Using a generalized-estimating-equation extension for the Cox proportional hazard model, we estimated the hazard ratio with an exchangeable covariance matrix to account for within-center clustering [22, 45]. For each of the 1000 sampled randomization schemes, the models were fitted to patient-level data from the trial population. We conducted unadjusted and another analysis adjusting for the randomization design (i.e., adjusted analyses using the constrained baseline characteristics by adding these variables into the model).

We stopped following patients on March 31, 2017, or earlier if they died. We summarized the hazard ratios as the mean with the 2.5th and 97.5th percentiles, corresponding to the hazard ratio estimate with a 95% confidence interval [46]. We expected to observe no between-group differences in the event rate of our primary outcome approximately 95% of the time (i.e., a nominal type I error of 5%). The use of 1000 randomizations allowed us to detect a type I error between 3.6% and 6.4% as not statistically different than 5%; we used a standard test based on the normal approximation to the binomial distribution as described by Rosner (1995) [47].

## Results

### Characteristics of cohorts

The population for randomization (*n*=5812) included all patients receiving in-center hemodialysis on April 1, 2013. The trial population (*n*=11,832) included patients receiving hemodialysis on April 1, 2014 (*n*=5410) and patients who started in-center hemodialysis during the 3-year trial period (*n*=6412). The trial population included 4415 patients (37%) in the population for randomization. The median (25th and 75th percentiles) number of patients in each center for the population for randomization was 61 (28, 105) and for the trial population was 131 (55, 227).

The population for randomization and the trial population differed on several baseline characteristics (Table 1 and Additional file 1). However, the differences were mainly attributed to the inherent differences between prevalent and new patients starting hemodialysis (e.g., length of time on dialysis, number of dialysis sessions in the prior year, healthcare service utilization, and general practitioner visits the preceding year.)

**Table.1:** Select baseline characteristics. The population for randomization included patients on hemodialysis as of April 1, 2013. The trial population included an open cohort of patients receiving in-center hemodialysis on April 1, 2014, or began receiving in-center hemodialysis during the trial period between April 1, 2014, and March 31, 2017

Baseline characteristic	Value	Population for randomization	Trial population
Centers	Number of centers (*n*, patients)	72 (*n*=5812)	72 (*n*=11,832)
Center Size ^a^	Mean ± standard deviation	81 (69)	164 (137)
	15 to 73 patients	42 (58%)	N/A
	74 to 131	19 (26%)	
	132 to 363	11 (15%)	
The composite outcome of CV-related death or major CV-related hospitalization ^b^	Historic rate per 100 person-years (cluster standard deviation)	10 (3.7)	11 (3.3)
	0.00 to 6.60	11 (15%)	7 (10%)
	6.70 to 9.90	14 (19%)	21 (29%)
	10.0 to 13.2	26 (36%)	25 (35%)
	13.3 to 23.1	21 (29%)	19 (26%)

^a^Population for randomization included patients that were on hemodialysis as of April 1, 2013, index date. The trial population included patients on hemodialysis as of April 1, 2014, and any patient who started in-center hemodialysis at one of the 72 participating centers during the 3-year trial period. Follow-up ended on March 31, 2017. The index date was the first date patients entered the respective cohort. N/A = not applicable because the center’s trial population had an open cohort, so the size was larger by design

^b^The composite outcome of cardiovascular-related death or hospitalization for myocardial infarction, ischemic stroke, and congestive heart failure

### Results from the principal component analysis

We subjected 29 of the 156 baseline characteristics to principal component analysis (Additional file 1: Appendix 4b). We retained ten principal components that accounted for 61% of the 29 baseline characteristics variance. Additional files 1: Appendix 6 and 7 show results from the principal component analysis.

### Randomization of hemodialysis centers

**Fig. 1:**
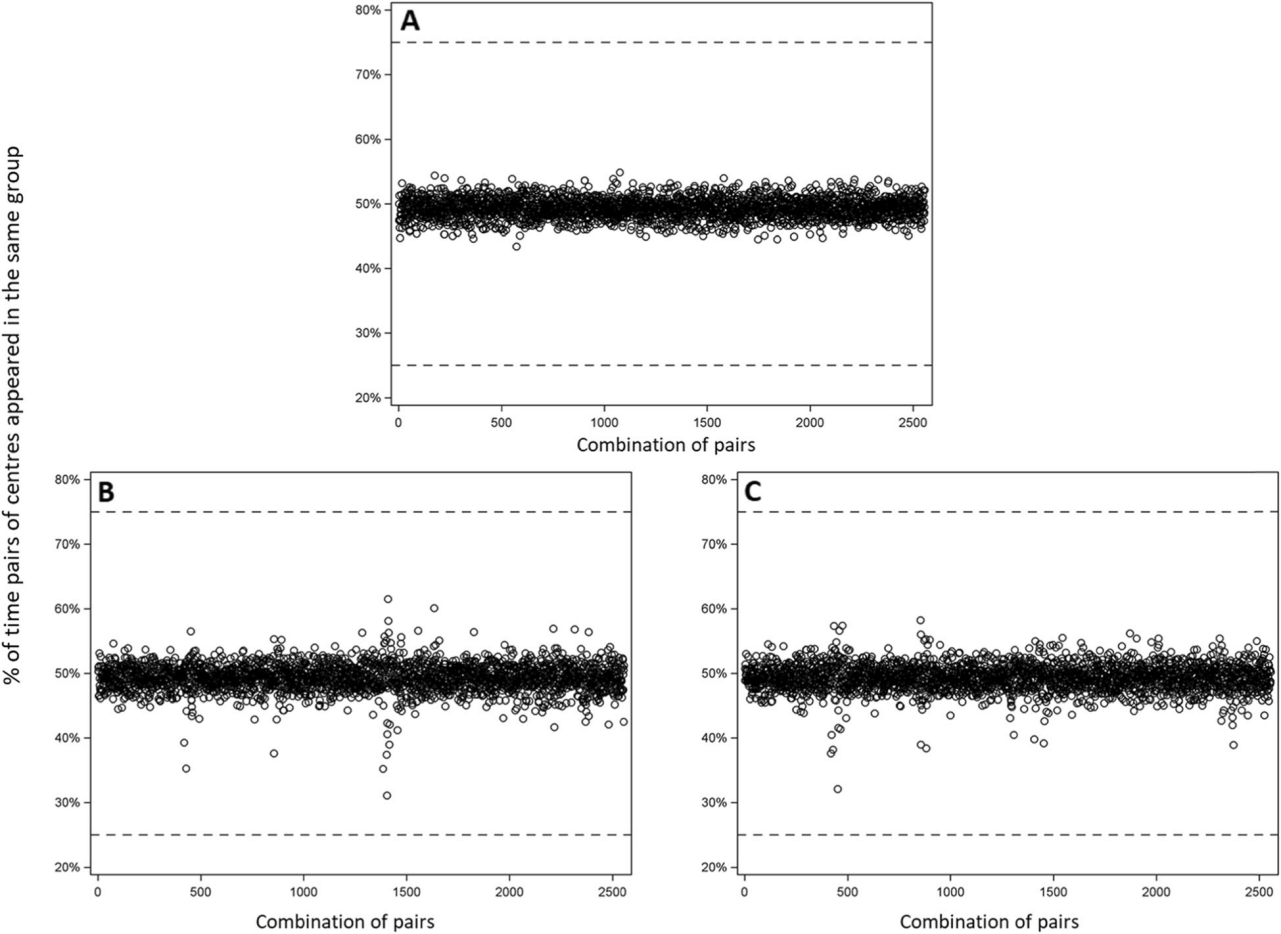
Percentage of time each pair of centers were randomly allocated to the same group (i.e., Center 1 with Center 2, Center 1 with Center 3, Center 1 with Center 4, ..., Center 71 with Center 72). There were a total of 2556 unique center pairs. **A** Centers randomly allocated without constraints (i.e., simple randomization) would appear in the same arm approximately 50% of the time. **B** Constraining on a subset of 11 prognostic baseline characteristics. **C** Constraining on ten principal components from a Principal Component Analysis. The horizontal dashed lines show center pairs (if any) allocated to the same arm 25% or 75% of the time [41]

Each of the 72 participating centers had an approximately 50% chance of being randomized to either trial arm (see Additional file 1: Appendix 8 for the process and hardware specification). However, we observed that some pairs of centers were allocated to different trial arms at a different probability than we might have expected if we had used simple randomization (Fig. 1A–C). In addition, these pairs of centers tended to be large and generally had over 225 patients.

### Balance of baseline characteristics

Table 2 shows the balance for a select set of baseline characteristics by the method of constraining. In the trial population, both sets of constrained variables were generally well balanced between the two arms, regardless of the randomization method. However, the constrained randomizations generally provided a slightly better balance. Additional file 1: Appendix 9 shows the percentage of times each of the baseline characteristics (from the trial population) were balanced across the 1000 randomization schemes for the three allocation methods. Table 3 shows a summary of the number of baseline characteristics balanced across randomization schemes. The trial population had at least 125 of 156 (80%) balanced baseline characteristics in 90% of simple randomization schemes. By comparison, the constrained methods always had slightly more balanced baseline characteristics (at least 85% of the 156 baseline characteristics were balanced in 90% of sampled allocations). Table 3 also shows the median (25th and 75th percentiles) number of balanced baseline characteristics across the 1000 sampled randomization schemes by allocation method.

**Table.2:** The percentage of times each of the baseline characteristics was balanced across each of the 1000 randomizations schemes in the trial population

Baseline characteristic	Value	Constrained randomization method		
		Unrestricted/simple	Prognostic baseline characteristics	Principal components
Center size	Mean ± standard deviation	32.9%	41.8%	38.7%
Composite outcome of CV-related death and major CV-related hospitalization	Rate (per 100 person-year)	32.5%	36.2%	33.5%
Age (years)	Mean ± standard deviation	95.3%	99.8%	99.2%
	< 65	97.8%	99.7%	99.9%
	65 to 74	100.0%	100.0%	100.0%
	75 to 84	100.0%	100.0%	100.0%
	85 to 105	99.5%	100.0%	99.9%
Sex	Male	100.0%	100.0%	100.0%
Living in a rural area	Yes	63.0%	84.2%	65.8%
Etiology for end-stage kidney disease	Diabetes	93.0%	94.5%	95.0%
	Glomerulonephritis/autoimmune diseases	96.3%	100.0%	99.5%
	Drug-induced nephropathy	100.0%	99.9%	100.0%
	Polycystic kidney disease	100.0%	100.0%	100.0%
	Renal vascular disease	97.5%	97.6%	96.7%
	Other	88.3%	91.9%	91.6%
Race	Asian	75.0%	81.3%	88.1%
	Black	73.4%	95.9%	91.9%
	White	45.6%	64.0%	90.2%
	Other	56.6%	65.7%	77.5%
	Unknown	93.2%	93.7%	93.6%
First dialysis modality	Home hemodialysis	100.0%	99.8%	99.9%
	In-center hemodialysis	97.8%	98.6%	99.9%
	Peritoneal dialysis	97.4%	98.7%	99.8%
First vascular access used at dialysis start	AV graft	99.9%	100.0%	100.0%
	Fistula	98.9%	99.1%	99.4%
	Catheter	93.5%	96.2%	99.4%
	PD catheter	98.8%	99.0%	100.0%
	Unknown	92.4%	93.8%	94.3%
Most recent vascular access before the index date	AV graft	98.7%	99.8%	98.9%
	Fistula	91.9%	94.8%	97.7%
	Catheter	89.9%	94.0%	97.4%
Patients 65+ years in ODB in the 6 months prior to index date	Yes	97.5%	99.3%	99.4%
Unique hypertensive drugs 6 months before the index date	Mean ± standard deviation	97.1%	99.9%	99.5%
Prescribed hypertensive drugs	Angiotensin-converting enzyme (ACE) inhibitors	99.4%	99.3%	99.5%
	Angiotensin II receptor blocker	90.7%	96.1%	96.9%
	Beta-blockers	99.7%	100.0%	99.9%
	Calcium channel blocker	98.1%	100.0%	99.6%
	Diuretics	91.9%	97.0%	95.6%
CABG/PCI	Yes	99.4%	99.5%	100.0%
Heart failure	Yes	96.8%	100.0%	99.8%
Diabetes mellitus	Yes	99.0%	100.0%	100.0%
Ischemic stroke	Yes	100.0%	100.0%	100.0%
Lower extremity amputation	Yes	99.9%	100.0%	100.0%
Lung disease (COPD)	Yes	99.0%	99.6%	100.0%
Myocardial infarction	Yes	99.2%	100.0%	100.0%
Major cancer	Yes	100.0%	100.0%	100.0%
Peripheral vascular disease	Yes	90.7%	97.2%	91.4%
Modified Charlson comorbidity Score	Mean ± standard deviation	96.8%	99.9%	100.0%
	2	97.7%	100.0%	100.0%
	3	100.0%	100.0%	100.0%
	4	100.0%	100.0%	100.0%
	5+	98.9%	100.0%	100.0%
Having a kidney transplant before the index date	Yes	100.0%	100.0%	100.0%
Number of hospital admissions in the year before the index date	Mean ± standard deviation	93.9%	98.4%	98.4%
	0	78.4%	76.4%	81.1%
	1 to 3	99.5%	99.6%	99.9%
	4 to 6	99.4%	99.6%	99.5%
	7 to 9	100.0%	99.9%	100.0%
	10+	92.1%	92.0%	94.6%
Long term care facility utilization in the year before the index date	Yes	81.3%	86.6%	86.1%
Time since the first date on dialysis (days)	Mean ± standard deviation	88.1%	94.0%	94.4%

**Table.3:** Summary of the balanced baseline characteristics for the trial population

Criteria	Constrained randomization method		
	Unconstrained/simple	Prognostic baseline characteristics	Principal components
**11 prognostic characteristics**^c^			
Number of constrained baseline characteristics that were balanced in all 1000 (100%) sampled allocations	0 of 11 (0%) ^a^	2 of 11 (18%)	2 of 11 (18%)
Number of constrained baseline characteristics that were balanced in at least 950 (95%) sampled allocations	6 of 11 (55%)	10 of 11 (91%)	7 of 11 (64%)
Number of constrained baseline characteristics that were balanced in at least 900 (90%) sampled allocations	8 of 11 (73%)	10 of 11 (91%)	9 of 11 (82%)
Median (25th and 75th percentile) number of baseline characteristics that were balanced across the 1000 selected randomization schemes	10 (9, 11) ^b^	11 (10, 11)	10 (10, 11)
**29 baseline characteristics used in the principal component analysis**^**d**^			
Number of constrained baseline characteristics that were balanced in all 1000 (100%) sampled allocations	8 of 29 (28%)	12 of 29 (41%)	12 of 29 (41%)
Number of constrained baseline characteristics that were balanced in at least 950 (95%) sampled allocations	19 of 29 (66%)	23 of 29 (79%)	25 of 29 (86%)
Number of constrained baseline characteristics that were balanced in at least 900 (90%) sampled allocations	24 of 29 (83%)	25 of 29 (86%)	26 of 29 (90%)
Median (25th and 75th percentile) number of baseline characteristics that were balanced across the 1000 selected randomization schemes	27 (26, 28)	28 (27, 28)	28 (28, 29)
**All 156 available baseline characteristics**			
Number of constrained baseline characteristics that were balanced in all 1000 (100%) sampled allocations	41 of 156 (26%)	46 of 156 (28%)	55 of 156 (35%)
Number of constrained baseline characteristics that were balanced in at least 950 (95%) sampled allocations	104 of 156 (67%)	115 of 156 (74%)	118 of 156 (76%)
Number of constrained baseline characteristics that were balanced in at least 900 (90%) sampled allocations	125 of 156 (80%)	132 of 156 (85%)	134 of 156 (86%)
Median (25th and 75th percentile) number of baseline characteristics that were balanced across the 1000 selected randomization schemes	147 (142, 150)	149 (146, 151)	150 (147, 151)

The trial population included patients on hemodialysis as of April 1, 2014, and new patients who started in-center hemodialysis during the 3-year follow-up. We conducted simple randomization without any restrictions

^a^For example, for simple randomization, 2 of the 11 chosen prognostic baseline characteristics were always balanced across 1000 randomly sampled allocation schemes

^b^For example, for simple randomization, 500 of 1000 allocation schemes had at least ten balanced baseline characteristics out of the 11 prognostic baseline characteristics. As such, there is a 50% probability that a randomly selected allocation will have at least 10 of the 11 prognostic baseline characteristics balanced and a 75% probability that at least 9 of the 11 prognostic baseline characteristics will be balanced

^c^Prognostic baseline characteristics: Constraining on a set of baseline characteristics that thought to be important a priori and included the following patient-level information: age at index date, living in a rural area, Black race, Modified Charlson comorbidity index, number of hospital visits in the previous 12 months, number of unique drugs the patient was prescribed in the 6 months before the index date, as well as history in the last 5 years of diagnosis for peripheral vascular disease, congestive heart failure, coronary artery disease, myocardial infarction, and number of nephrology consults in the previous 12 months before the index date

^d^Results are shown for the 29 baseline characteristics included in the principal component analysis. We did not include any cluster-level baseline characteristics in the constraining process

### Cardiovascular-related death or major cardiovascular-related hospitalization

We followed patients for an average of 1.7 years, and there were 2260 events over the 3-year follow-up. The event rate of the primary outcome was 11 per 100 person-years. Table 4 shows the results from the unadjusted and adjusted analyses for simple and covariate-constrained randomization methods. Across the 1000 simple randomization schemes for the trial population, the mean unadjusted hazard ratio (2.5th and 97.5th percentile) was 1.01 (0.87, 1.16), and 5.9% of allocation schemes produced a confidence interval for the hazard ratio that did not contain the null value of 1.00. Compared to simple randomizations, constrained randomizations had similar unadjusted hazard ratios, with slightly narrower 95% confidence intervals. The type I error tended to be somewhat lower than the nominal level for some constrained methods than the unconstrained approach.

**Table.4:** Mean hazard ratio (2.5th and 97.5th percentiles) for the composite outcome during a 3-year follow-up of patients on in-center hemodialysis

Baseline characteristics adjusted in the analysis	Mean HR	Width of CI ^**a**^	Type 1 error^**c**^
	(2.5th and 97.5th percentile)		
**Unadjusted analyses**			
Simple (i.e., unconstrained) randomization	1.01 (0.87, 1.16)	0.280	5.9%^e^
Constrained on a minimal set of baseline characteristics^b^	1.00 (0.89, 1.12)	0.233	3.2%
Constrained on a minimal set of baseline characteristics^b^ and historical rate of the primary outcome	1.00 (0.88, 1.13)	0.250	4.4%^e^
Constrained on a minimal set of baseline characteristics^b^ and cluster size at time of randomization	1.00 (0.88, 1.14)	0.260	5.2%^e^
Constrained on a minimal set of baseline characteristics^b^, historical rate of the primary outcome, and cluster size at time of randomization	1.00 (0.88, 1.13)	0.247	4.5%^e^
Constrained on 10 principal components	1.01 (0.89, 1.12)	0.234	3.3%
Constrained on 10 principal components and historic rate of primary outcome	1.00 (0.88, 1.14)	0.261	5.2%^e^
Constrained on 10 principal components and cluster size at time of randomization	1.00 (0.87, 1.14)	0.264	4.1%^e^
Constrained on ten principal components, the historical rate of the primary outcome, and cluster size at time of randomization	1.00 (0.89, 1.13)	0.239	3.1%
**Adjusted for constrained baseline characteristics**^**d**^			
Constrained on a minimal set of baseline characteristics^b^	1.00 (0.89, 1.12)	0.232	8.6%
Constrained on a minimal set of baseline characteristics^b^ and historical rate of the primary outcome	1.00 (0.89, 1.12)	0.223	8.3%
Constrained on a minimal set of baseline characteristics^b^ and cluster size at time of randomization	1.00 (0.89, 1.11)	0.221	9.8%
Constrained on a minimal set of baseline characteristics^b^, historical rate of the primary outcome, and cluster size at time of randomization	1.00 (0.90, 1.11)	0.216	9.6%
Constrained on 10 principal components	1.00 (0.90, 1.11)	0.203	5.2%^e^
Constrained on 10 principal components and historic rate of primary outcome	1.00 (0.90, 1.11)	0.201	6.0%^e^
Constrained on 10 principal components and cluster size at time of randomization	1.00 (0.90, 1.11)	0.203	6.3%^e^
Constrained on ten principal components, the historical rate of the primary outcome, and cluster size at time of randomization	1.00 (0.91, 1.11)	0.201	6.4%^e^

All randomization methods had 1000 randomization schemes. The cohort included patients on dialysis as of April 1, 2014, and any patient who started in-center hemodialysis at one of the 72 participating centers during the 3-year follow-up

*HR* hazard ratio, *width of CI* width of confidence interval (i.e., upper minus lower confidence limit)

^**a**^The confidence interval’s width may not equal the difference between the lower and upper confidence limits because of rounding

^b^Included patient-level information: age, living in a rural area, Black race, Modified Charlson comorbidity index, number of hospital visits in the previous 12 months, number of unique drugs the patient was prescribed in the 6 months before the index date, as well as history in the last 5 years of diagnosis for peripheral vascular disease, congestive heart failure, coronary artery disease, myocardial infarction, and number of nephrology consults in the previous 12 months before the index date

^c^Type 1 error in the various constrained scenarios. Note: The nominal type 1 error is 5%. The observed type 1 error was within an “acceptable range” if it fell within the 95% confidence interval of the nominal value (i.e., between 3.6% and 6.4%)

^**d**^Adjusted analyses included baseline characteristics used in the constraining process

^e^An acceptable type 1 error was observed for this method (i.e., between 3.6% and 6.4%)

Adjusted analyses for the constrained methods produced narrower confidence intervals than the unadjusted analyses. However, the type I error was within the acceptable range only when models adjusted for the ten principal components; the type I error was outside the expected range for all other adjusted analyses. We also explored the results when adjusting for aggregate-level baseline characteristics as used in the randomization, which aligned with the results when we adjusted for individual-level variables (results not shown).

## Discussion

This empirical study presented an example of using historical data to conduct covariate-constrained randomization that balances baseline characteristics for a parallel, two-group, cluster-randomized trial. We showed that constraining the random allocation using a historical cohort (i.e., a population for randomization) provides a better balance on baseline characteristics than simple randomization. However, we randomized a moderate number of clusters, and the magnitude of benefit was modest. Our results also suggested that model-based adjustment for the constrained variables produced treatment effects with the nominal type I error that is narrower than those produced with simple randomization. However, researchers should constrain prognostic variables and adjust for the constrained variables at the analysis stage; otherwise, the type I error might deviate from the nominal level described in previous reports [1, 9, 11, 12, 17, 18].

In a review of 300 CRTs published between 2000 and 2008, Wright et al. found significant discrepancies between the restricted randomization used at the design stage and covariate adjustments at the analysis stage [48]. Wright et al. identified 174 CRTs that used design-based restricted randomization [48]. However, only 30 (17.2%) of these studies reported an adjusted analysis for all the constrained variables.

From an analysis perspective, the analysis should account for the design that uses covariate-constrained randomization [1, 9, 11, 12]. Otherwise, the type I error may deviate from the nominal level because clusters with highly correlated outcomes get separated into different treatment arms (as observed in Fig. 1B, C) [9]. Splitting correlated clusters into different treatment arms tends to (i) lower the type I error below the nominal level (in the unadjusted analyses), and (ii) decrease power slightly, although we might still expect substantial gains in power due to the assurance of balance on prognostic baseline characteristics [9, 49]. Several analytical techniques can test for treatment effects and take into account the study design. These methods include mixed-effects models, bias-corrected generalized estimating equations, and randomization-based permutation tests.

In our motivating example, we used an analysis for the time-to-first event. In contrast, previous studies have focused their investigations primarily on continuous or binary outcomes [1, 9, 11, 12]. Our results add to this literature showing a generalized estimating equation-based approach can yield results that maintain the nominal type I error after adjusting for the covariate-constrained design.

This study has some limitations. First, the trial population included a large percentage of patients (37%) included in the population for randomization. Thus, our results may not apply to other designs, for example, CRTs where the *population for randomization* and the trial population are the same or settings where cluster- and patient-level profiles change rapidly over time. Second, some historical data may lag by more than 1 year; thus, these results may not apply for *populations at randomization* less than or more than a year old. Third, our example cohort randomized a moderately large number of clusters; a previous review reported that 75% of published CRTs randomized fewer than 52 clusters. Covariate-constrained randomization may provide a better baseline balance compared to simple randomization when there are fewer clusters. Finally, our secondary objective does not constitute a formal test of the type I error. Computer simulations with more control over the generated data would be better suited. As such, the reader should interpret these results cautiously.

## Conclusions and guidance for future trials

Although covariate-constrained randomization approaches used in this setting had modest improvement for balance, there may be substantial improvements in statistical power [12]. We propose the following recommendations (Table 5) for CRTs based on the empirical comparisons presented in this paper and other published literature. It is worth noting that these recommendations are based on a single setting, and while we anticipate similar findings in different contexts, a more formal statistical comparison would be beneficial.

1. Identify prognostic variables a priori using background literature, historical data, or previous trials. Previous work for individual-level randomized controlled trials showed increases in statistical power when analyses prespecified covariates strongly associated with the outcome. The adjusted covariates had a more considerable impact on statistical power when the prevalence was moderate to high (between 10% and 50%) [19, 50–52].
2. Researchers should consider generating all (or at least 1000) simple randomizations to identify baseline characteristics that are always or almost always balanced (e.g., >95% of the time) between treatment arms. There would be no need to include these baseline characteristics in the constraining process; however, researchers can have these variables in the model-based adjustment to improve the estimates’ precision. Importantly, all prognostic variables should be specified a priori [52].
3. Carefully consider the number of baseline characteristics used during the constraining process. Evidence from our study (and previous simulation studies) showed that over-constraining could result in clusters with highly correlated outcomes having a lower probability of being included in the same trial arm. Thus, over-constraining can lead to a type I error below the nominal level and slightly decrease power [9, 49].
4. Researchers can use a dimensionality-reduction method (e.g., principal component analysis) to reduce many dimensions of the prognostic variables to several criterion variables used in the constrained randomization process [53]. As above, all analyses should account for the dimensionality-reduction criterion at the analytic stage.
5. While the constraining process utilizes aggregate patient-level and cluster-level data, investigators should consider missingness when constraining the randomization on these variables. When appropriate, variables with missing data should be imputed before aggregating the variable at the cluster level [54].
6. Researchers should consider constraining the randomization space to the 10% best allocations. Furthermore, researchers should enumerate all possible randomization schemes when fewer than 20 clusters or at least 100,000 randomization schemes [12].

**Table.5:** Guidance for conducting covariate-constrained randomization

1. Identify prognostic baseline characteristics a priori using background literature, historical data, or previous trials.	
2. Generate all (or at least 1000) simple randomizations to identify baseline characteristics that are always balanced between treatment arms (e.g., ≥95% of the time).	
3. Carefully consider the number of variables added to the constraining process or consider using a dimensionality-reduction method for many variables (e.g., principal component analysis).	
4. Consider the amount of missingness of constrained baseline characteristics prior to randomization.	
5. Enumerate all possible allocation schemes when there are fewer than 20 clusters or at least 100,000 allocations otherwise.	

## Supplementary Information

**Additional file 1: Appendix 1.** Common data sources used for population-based studies. **Appendix 2.** Complete list of 156 Baseline characteristics for the randomization and trial population cohorts. **Appendix 3.** Randomization of the 72 clusters using PROC PLAN in SAS. **Appendix 4.** a Prognostic baseline characteristics that were thought to be relevant a priori or correlated with the outcome from previous literature. b Baseline characteristics from the Population for Randomization that were subjected to principal component analysis. **Appendix 5.** Algorithm for capturing primary composite outcome. **Appendix 6.** Results from Principal component analysis (PCA). **Appendix 7.** We used the principal axis method to extract the principal components. A varimax (orthogonal) rotation followed the principal axis method. Only the first ten components displayed eigenvalues greater than 1 (see **Appendix 6**), and the results of a scree test also suggested that only the first ten components were meaningful. Therefore, we retained the first ten components for rotation. **Appendix 8.** Hardware specification and optimization for running the constrained randomization process. **Appendix 9.** The percentage of times baseline characteristics were balanced across 1000 randomization schemes for the three techniques.

## Data Availability

While data sharing agreements prohibit ICES from making our study dataset publicly available, access may be granted to those who meet prespecified criteria for confidential access, available at www.ices.on.ca/das. In addition, the full dataset creation plan and underlying analytic code can be requested from the authors on the understanding that the computer programs may rely upon coding templates or macros that are unique to ICES and are therefore either inaccessible or may require modification.
