# Supplementary material for: Simple compared to covariate-constrained randomization methods in balancing baseline characteristics: a case study of randomly allocating 72 hemodialysis centers in a cluster trial
Source: Trials. 2021 Sep 15;22:626. doi: 10.1186/s13063-021-05590-1 (PMC8444397; doi:10.1186/s13063-021-05590-1)
Supplement: Supplementary file 1 — Additional file 1: Appendix 1. Common data sources used for population-based studies. Appendix 2. Complete list of 156 Baseline characteristics for the randomization and trial population cohorts. Appendix 3. Randomization of the 72 clusters using PROC PLAN in SAS. Appendix 4. a Prognostic baseline characteristics that were thought to be relevant a priori or correlated with the outcome from previous literature. b Baseline characteristics from the Population for Randomization that were subjected to principal component analysis. Appendix 5. Algorithm for capturing primary composite outcome. Appendix 6. Results from Principal component analysis (PCA). Appendix 7. We used the principal axis method to extract the principal components. A varimax (orthogonal) rotation followed the principal axis method. Only the first ten components displayed eigenvalues greater than 1 (see Appendix 6), and the results of a scree test also suggested that only the first ten components were meaningful. Therefore, we retained the first ten components for rotation. Appendix 8. Hardware specification and optimization for running the constrained randomization process. Appendix 9. The percentage of times baseline characteristics were balanced across 1000 randomization schemes for the three techniques. [file 13063_2021_5590_MOESM1_ESM.docx]

**Appendix 1**: Common Data Sources used for Population-Based Studies

| **Database (Source)** | **Description** | **Key Data Variables** |
| --- | --- | --- |
| **Health Services** | | |
| Discharge Abstract Database (CIHI) | Hospital discharge abstracts for acute, chronic, and rehabilitative care (1988 onward) | Diagnoses; Procedures; Comorbidities; Length of Stay |
| National Ambulatory Care Reporting System (CIHI) | ED visits, same-day surgery, outpatient clinics (e.g., dialysis, cancer clinics) (2002 onward) | Reason for the visit; Triage level; Interventions; Mode of arrival |
| Ontario Drug Benefit Database (MOHLTC) | Claims for prescribed drugs covered by the Ontario Drug Formulary for adults aged 65+ and those receiving social assistance (1990 onward) | Drug ID number; Drug quantity; Cost |
| Ontario Health Insurance Plan (MOHLTC) | Reimbursement claims made by fee-for-service physicians and community-based labs (1991 onward) | Service provided; Diagnosis codes; Fee paid; Physician specialty |
| **Registry** | | |
| Canadian Organ Replacement Register (CIHI) | Collects and records the incidence, prevalence, treatment changes, and outcomes of all chronic dialysis and solid organ transplant patients in Canada. Data is collected by voluntary completion of survey forms for each patient at dialysis initiation and at yearly follow-up (2001 onward) | Hemodialysis start; vascular access use; nephrology referral; comorbid and baseline conditions |
| Ontario Renal Reporting System | Collects and records the incidence, prevalence, treatment changes, and outcomes of all chronic dialysis and solid organ transplant patients in Canada. Data collected is mandated by the Ontario Renal Network for each patient at dialysis initiation and yearly follow-up (2010 onward) | Hemodialysis start; vascular access use; nephrology referral; comorbid and baseline conditions |
| **Population and Demographics** | | |
| Registered Persons Database (MOHLTC) | Demographic information about all Ontarians that ever had an Ontario Health Card Number. (1990 onward) | Date of birth; Date of death; Sex; Geographic information |
| Office of the Registrar General- Deaths (ORGD) | ORGD is an annual dataset containing information on all deaths registered in Ontario starting on January 1, 1990. | **Note**: Information on cause of death lags other variables by ~2 years. |
| **Care Providers** | | |
| ICES Physicians Database | This data set contains yearly information about all physicians in Ontario (1992 onward) | Annual demographics; Specialization; Workload |

MOHTC: Ministry of Health and Long-term Care, CIHI – Canadian Institutes for Health Information

**Appendix 2**: Complete list of 156 Baseline characteristics for the randomization and trial population cohorts

| **Baseline characteristic** | **VALUE** | **Population for Randomization** | **Trial Population** |
| --- | --- | --- | --- |
| Center Size ^1^ | Mean ± Standard deviation | 81 (69) | 164 (137) |
| The composite outcome of CV-related death and major CV-related hospitalization ^2^ | Historic rate (per 100 person-years) | 10 | 10 |
| Age (years) | Mean ± Standard deviation | 67 (15) | 66 (15) |
|  | < 65 | 2348 (40%) | 4913 (42%) |
|  | 65 to 74 | 1450 (25%) | 3060 (26%) |
|  | 75 to 84 | 1464 (25%) | 2853 (24%) |
|  | 85 to 105 | 550 (9%) | 1006 (9%) |
| Sex | Male | 3373 (58%) | 7069 (60%) |
| Living in a rural area | Yes | 359 (6%) | 809 (7%) |
| Neighbourhood Income Quintile | 1 | 1682 (29%) | 3748 (32%) |
|  | 2 | 1317 (23%) | 2665 (23%) |
|  | 3 | 1070 (18%) | 2132 (18%) |
|  | 4 | 960 (17%) | 1799 (15%) |
|  | 5 | 740 (13%) | 1467 (12%) |
|  | Missing | 43 (1%) | 21 (0%) |
| Etiology for ESKD | Diabetes | 2194 (38%) | 4472 (38%) |
|  | Glomerulonephritis/autoimmune diseases | 882 (15%) | 1575 (13%) |
|  | Drug-induced nephropathy | 83 (1%) | 159 (1%) |
|  | Polycystic kidney disease | 229 (4%) | 426 (4%) |
|  | Renal vascular disease | 1115 (19%) | 1852 (16%) |
|  | Other | 738 (13%) | 2221 (19%) |
|  | Unknown | 571 (10%) | 1127 (10%) |
| Race | Asian | 475 (8%) | 854 (7%) |
|  | Black | 562 (10%) | 1022 (9%) |
|  | White | 3698 (64%) | 7598 (64%) |
|  | Other | 1038 (18%) | 2173 (18%) |
|  | Unknown | 39 (1%) | 185 (2%) |
| First dialysis modality | Home hemodialysis | 33 (1%) | 84 (1%) |
|  | In-center hemodialysis | 5215 (90%) | 10529 (89%) |
|  | Peritoneal dialysis | 564 (10%) | 1219 (10%) |
| Initial vascular access used at dialysis start | Arteriovenous graft | 103 (2%) | 198 (2%) |
|  | Arteriovenous fistula | 1044 (18%) | 1999 (17%) |
|  | Catheter | 3927 (68%) | 8157 (69%) |
|  | Peritoneal catheter | 456 (8%) | 1092 (9%) |
|  | Unknown | 282 (5%) | 386 (3%) |
| Most recent vascular access before the index date | Arteriovenous graft | 223 (4%) | 376 (3%) |
|  | Arteriovenous fistula | 2159 (37%) | 3461 (29%) |
|  | Catheter | 3376 (58%) | 7544 (64%) |
| Patients < 65 years in Ontario Drug benefit in the six months before the index date | Yes | 1596 (27%) | 3431 (29%) |
| Patients 65+ years in Ontario Drug Benefit in the six months before the index date | Yes | 3398 (58%) | 6765 (57%) |
| Angiotensin-converting enzyme (ACE) inhibitors | Yes | 1157 (26%) | 2199 (22%) |
| Angiotensin II Receptor Blocker | Yes | 1258 (28%) | 2413 (24%) |
| Alpha-Blockers | Yes | 408 (9%) | 1073 (11%) |
| Benzodiazepine | Yes | 1060 (24%) | 1884 (18%) |
| Beta-Blockers | Yes | 2649 (59%) | 5604 (55%) |
| Centrally Acting Antiadrenergic | Yes | 143 (3%) | 338 (3%) |
| Calcium Channel Blocker | Yes | 2392 (53%) | 5453 (53%) |
| Diuretics | Yes | 1612 (36%) | 4242 (42%) |
| Midodrine | Yes | 250 (6%) | 517 (5%) |
| Vasodilators | Yes | 226 (5%) | 778 (8%) |
| Anti-psychotics | Yes | 309 (7%) | 657 (6%) |
| Anti-depressants | Yes | 1270 (28%) | 2531 (25%) |
| Number of unique hypertensive prescriptions in the six months before the index date | Mean ± Standard deviation | 2 (2) | 2 (2) |
| Number of hypertensive subclasses prescribed in the six months before the index date | Mean ± Standard deviation | 2 (1) | 2 (2) |
| Abdominal aortic aneurysm repair/aortic bypass | Yes | 34 (1%) | 78 (1%) |
| Atrial Fibrillation/Flutter | Yes | 914 (16%) | 2045 (17%) |
| Acute Kidney Injury in the six months before the index date | Yes | 1431 (25%) | 3934 (33%) |
| Alcoholism | Yes | 97 (2%) | 256 (2%) |
| Lower extremity amputation | Yes | 298 (5%) | 554 (5%) |
| Arrhythmia | Yes | 1431 (25%) | 3119 (26%) |
| CABG/PCI | Yes | 1234 (21%) | 2612 (22%) |
| Coronary Artery Disease (with angina) | Yes | 3541 (61%) | 6861 (58%) |
| Heart failure | Yes | 2862 (49%) | 6177 (52%) |
| Diabetes mellitus | Yes | 3402 (59%) | 7244 (61%) |
| Dementia | Yes | 892 (15%) | 1830 (15%) |
| Depression | Yes | 1528 (26%) | 3161 (27%) |
| Having any type of Fracture | Yes | 604 (10%) | 1092 (9%) |
| Fracture of the Humerus | Yes | 26 (0%) | 48 (0%) |
| Fracture of the Pelvis | Yes | 80 (1%) | 130 (1%) |
| Fracture of the Femur | Yes | 136 (2%) | 267 (2%) |
| Fracture of the Hip | Yes | 302 (5%) | 526 (4%) |
| Fracture of the wrist | Yes | 237 (4%) | 445 (4%) |
| Hypertension | Yes | 5629 (97%) | 11453 (97%) |
| Hypotension | Yes | 669 (12%) | 1382 (12%) |
| Ischemic Stroke | Yes | 261 (4%) | 551 (5%) |
| Subarachnoid Hemorrhage | Yes | 6 (0%) | 20 (0%) |
| Liver disease | Yes | 697 (12%) | 1521 (13%) |
| Lung disease (COPD) | Yes | 2276 (39%) | 4666 (39%) |
| Myocardial infarction | Yes | 1454 (25%) | 2909 (25%) |
| Malignancy (excluding skin cancer) | Yes | 2339 (40%) | 5001 (42%) |
| Major Cancer | Yes | 841 (14%) | 1803 (15%) |
| Other Serious Illness that could shorten life expectancy to less than five years | Yes | 845 (15%) | 1949 (16%) |
| Peripheral vascular disease | Yes | 1699 (29%) | 3055 (26%) |
| Having a kidney transplant before the index date | Yes | 71 (1%) | 148 (1%) |
| Smoker | Yes | 733 (13%) | 1564 (13%) |
| Syncope | Yes | 248 (4%) | 494 (4%) |
| Venous thromboembolism | Yes | 344 (6%) | 779 (7%) |
| Stroke/Transient ischemic attack (TIA) | Yes | 1018 (18%) | 2029 (17%) |
| Body mass index | Mean ± Standard deviation | 28 (8) | 28 (8) |
|  | Underweight | 280 (5%) | 414 (3%) |
|  | Normal | 1816 (31%) | 3526 (30%) |
|  | Overweight | 1599 (28%) | 3376 (29%) |
|  | Obese I | 913 (16%) | 2040 (17%) |
|  | Obese II | 433 (7%) | 962 (8%) |
|  | Obese III | 348 (6%) | 799 (7%) |
|  | Missing | 423 (7%) | 715 (6%) |
| Modified Charlson comorbidity Score | Mean ± Standard deviation | 4 (2) | 4 (2) |
|  | 2 | 1931 (33%) | 3631 (31%) |
|  | 3 | 519 (9%) | 1028 (9%) |
|  | 4 | 1382 (24%) | 2883 (24%) |
|  | 5+ | 1980 (34%) | 4290 (36%) |
| Abdominal/Renal ultrasound | Yes | 5064 (87%) | 10611 (90%) |
| Chest x-ray | Yes | 5690 (98%) | 11567 (98%) |
| Coronary angiogram | Yes | 1267 (22%) | 2540 (21%) |
| Coronary revascularization | Yes | 681 (12%) | 1418 (12%) |
| Echocardiography | Yes | 5168 (89%) | 10440 (88%) |
| Holter monitoring | Yes | 1583 (27%) | 3401 (29%) |
| Stress test | Yes | 3442 (59%) | 6969 (59%) |
| Carotid endarterectomy | Yes | 18 (0%) | 44 (0%) |
| Number of cardiology visits in the year prior to the index date | Mean ± Standard deviation | 3 (5) | 5 (7) |
|  | 0 | 1547 (27%) | 2283 (19%) |
|  | 1 to 3 | 2465 (42%) | 4666 (39%) |
|  | 4 to 6 | 977 (17%) | 2196 (19%) |
|  | 7 to 9 | 352 (6%) | 1046 (9%) |
|  | 10+ | 471 (8%) | 1641 (14%) |
| Number of general practitioner visits in the year before the index date | Mean ± Standard deviation | 9 (13) | 13 (16) |
|  | 0 | 1547 (27%) | 2283 (19%) |
|  | 1 to 3 | 2465 (42%) | 4666 (39%) |
|  | 4 to 6 | 977 (17%) | 2196 (19%) |
|  | 7 to 9 | 352 (6%) | 1046 (9%) |
|  | 10+ | 471 (8%) | 1641 (14%) |
| Number of nephrology consults in the year prior to the index date | Mean ± Standard deviation | 8 (14) | 12 (17) |
|  | 0 | 1547 (27%) | 2283 (19%) |
|  | 1 to 3 | 2465 (42%) | 4666 (39%) |
|  | 4 to 6 | 977 (17%) | 2196 (19%) |
|  | 7 to 9 | 352 (6%) | 1046 (9%) |
|  | 10+ | 471 (8%) | 1641 (14%) |
| Number of days spent in the hospital in the year prior to the index date | Mean ± Standard deviation | 11 (28) | 17 (31) |
|  | 0 | 1547 (27%) | 2283 (19%) |
|  | 1 to 3 | 2465 (42%) | 4666 (39%) |
|  | 4 to 6 | 977 (17%) | 2196 (19%) |
|  | 7 to 9 | 352 (6%) | 1046 (9%) |
|  | 10+ | 471 (8%) | 1641 (14%) |
| Number of hospitalization visits in the year prior to the index date | Mean ± Standard deviation | 1 (1) | 1 (1) |
|  | 0 | 1547 (27%) | 2283 (19%) |
|  | 1 to 3 | 2465 (42%) | 4666 (39%) |
|  | 4 to 6 | 977 (17%) | 2196 (19%) |
|  | 7 to 9 | 352 (6%) | 1046 (9%) |
|  | 10+ | 471 (8%) | 1641 (14%) |
| Number of emergency department visits in the year before the index date | Mean ± Standard deviation | 2 (3) | 3 (3) |
|  | 0 | 1547 (27%) | 2283 (19%) |
|  | 1 to 3 | 2465 (42%) | 4666 (39%) |
|  | 4 to 6 | 977 (17%) | 2196 (19%) |
|  | 7 to 9 | 352 (6%) | 1046 (9%) |
|  | 10+ | 471 (8%) | 1641 (14%) |
| Number of days spent in long-term care in the year prior to the index date | Mean ± Standard deviation | 37 (164) | 25 (133) |
| Long term care facility utilization in the year before the index date | Yes | 568 (10%) | 1082 (9%) |
| Number of dialysis sessions in the year prior to the index date | Mean ± Standard deviation | 140 (36) | 107 (48) |
| Time since the first date on dialysis (days) | Mean ± Standard deviation | 1847 (1836) | 1327 (1782) |
| Height (cm) prior to starting dialysis | Mean ± Standard deviation | 175 (63) | 170 (42) |
| Weight (kg) prior to starting dialysis | Mean ± Standard deviation | 87 (71) | 83 (50) |
| Urea test result before starting dialysis | Mean ± Standard deviation | 33 (21) | 32 (22) |
| Hemoglobin test results prior to starting dialysis | Mean ± Standard deviation | 97 (23) | 97 (35) |
| Creatinine test result prior to starting dialysis | Mean ± Standard deviation | 632 (342) | 634 (331) |
| eGFR using CKD EPI | Mean ± Standard deviation | 9 (6) | 9 (6) |
| Serum albumin test result prior to starting dialysis | Mean ± Standard deviation | 32 (7) | 32 (7) |

^1^ Population for Randomization included patients that were on hemodialysis as of April 1, 2013, and Trial Population included patients that were on hemodialysis as of April 1, 2014, and any patient that started in-center hemodialysis at one of the 72 participating centers during the three-year follow-up. ^2^ Composite outcome of cardiovascular-related death or hospitalization for myocardial infarction, ischemic stroke, congestive heart failure. ^3^ Percentage presented only for patients eligible to receive the Ontario Drug Benefit (ODB) plan in the six months before the index date (i.e., 4494 for Randomization Cohort and 10196 for the Trial cohort). CABG/PCI = Coronary artery bypass grafting (CABG) / percutaneous coronary intervention; GN = Glomerulonephritis; ESKD=End-stage kidney disease.

**Appendix 3**: Randomization of the 72 clusters using PROC PLAN in SAS.

***********************************;

**Creating randomizations Schemes**;

***********************************;

**proc** **plan** seed=**14424**;

factors set=**300000** group=**72** / noprint;

output out=a;

**run**;

**proc** **sort** data=a;

by set;

**run**;

**data** b;

label Set="The allocation number"

Center_Number="Unique Cluster Number"

Arm="Treatment arm"

;

set a;

by set;

retain Center_Number;

Arm=(group LE **36**);

if first.set then

Center_Number=**0**;

Center_Number=Center_Number+**1**;

drop group;

**run**;

**Appendix 4a**: Prognostic baseline characteristics that were thought to be relevant a priori or correlated with the outcome from previous literature

Prognostic factors included the following patient-level information: age at index date, living in a rural area, Black race, Modified Charlson comorbidity index, number of hospital visits in the previous 12 months, number of unique drugs the patient was prescribed in the six months before the index date, as well as history in the last five years of diagnosis for peripheral vascular disease, congestive heart failure, coronary artery disease, myocardial infarction, number of nephrology consults in the previous 12 months before the index date. We also included cluster-level baseline characteristics such as center size and the historical rate of the primary composite outcome of cardiovascular-related death or hospitalization for myocardial infarction, ischemic stroke, or heart failure.

We excluded several prognostic factors from above (e.g., diabetes) because these baseline characteristics were almost always balanced in the non-constrained setting (See Appendix 9). As such, constraining on these baseline characteristics would not have influenced the results for the constrained randomization.

**Appendix 4b**: The 156 baseline characteristics from the Population for Randomization were subjected to a principal component analysis using one as prior communality estimates; communalities refers to the estimate of the variances for the principal components.^1^ We dropped 127 baseline characteristics that loaded on more than one component because these baseline characteristics are not pure measures of any single construct. Thus, 29 baseline characteristics were included in the analysis. We used the principal axis method to extract the components, followed by a varimax (orthogonal) rotation.^2^ We retained principal components for rotation when the eigenvalues were greater than one. In interpreting the rotated factor pattern, an item loaded on a given component if the factor loading was equal to or greater than 40% for that component and less than 40% for the others.

**Appendix 5**: Algorithm for capturing primary composite outcome

| **Outcome** | **Algorithm** | **Position of code** | **Performance** |
| --- | --- | --- | --- |
| Cardiovascular-related death ^₳, ¥^ | ORGD: Leading Cause of Death  LCD_33 = Chronic rheumatic heart disease  LCD_34 = Hypertensive disease LCD_35 = Ischemic heart disease LCD_36 = Pulmonary heart disease and related LCD_37 = Nonrheumatic valve disorders LCD_38 = Cardiomyopathy LCD_39 = Cardiac arrest LCD_40 = Cardiac arrhythmias LCD_41 = Heart failure and complications, ill-defined heart disease LCD_42 = Cerebrovascular diseases LCD_43 = Atherosclerosis LCD_44 = Aortic aneurysm and dissection | N/A | Not available |
| Cardiovascular-related death | **ICD-10:**  I00 - I78  AND  Discharge disposition of '07' or death in the Registered Persons Database during the hospital stay | Primary Diagnosis | RPDB has an accuracy of 99% for capturing death ^3^ |
| Hospital admission with ischemic stroke | ***ICD-10:***  I63 (excl. I63.6), I64, H341 | Primary Diagnosis | PPV= 85% ^4,5^ |
| Hospital admission with myocardial infarction | ***ICD-10:***  I21, I22 | Primary Diagnosis | Sn= 89%, PPV= 87% ^6^ |
| Hospital admission with heart failure | **ICD-10:**  I50 | Primary Diagnosis | Sn=61% , Sp=98%,  PPV=66%^7^ |

Abbreviations: ICD = International Classification of Disease; OHIP = Ontario Health Insurance Plan; Dischdisp=Discharge disposition; Sn=Sensitivity; PPV= Positive Predictive Value; LCD=Leading Cause of Death; ORGD=Office of Registrar General - Deaths.

₳ Due to the time lag in data capture, deaths from ORGD will only capture events for the follow-up period between April 3, 2017, and December 31, 2020. These events capture both in- and out-of-hospital cardiovascular-related deaths. For the remaining study period, we will only be able to capture in-hospital deaths using ICD-10 codes.

¥ Personal communication with Dr. Jack Tu, who was part of a working group conducting validation of this outcome using existing Ontario clinical trial data as the reference standard.

**Appendix 6:** Results from Principal component analysis (PCA).

| **Eigenvalues of the Correlation Matrix** | | | |
| --- | --- | --- | --- |
| Principal component | **Eigenvalue**** | **The proportion of variance explained** | **Cumulative variance explained** |
| **1** | 3.67 | 0.13 | 0.13 |
| **2** | 2.19 | 0.08 | 0.20 |
| **3** | 2.04 | 0.07 | 0.27 |
| **4** | 1.72 | 0.06 | 0.33 |
| **5** | 1.60 | 0.06 | 0.39 |
| **6** | 1.53 | 0.05 | 0.44 |
| **7** | 1.33 | 0.05 | 0.49 |
| **8** | 1.30 | 0.05 | 0.53 |
| **9** | 1.13 | 0.04 | 0.57 |
| **10** | 1.03 | 0.04 | 0.61 |
| **11** | 0.99 | 0.03 | 0.64 |
| **12** | 0.89 | 0.03 | 0.67 |
| **13** | 0.83 | 0.03 | 0.70 |
| **14** | 0.82 | 0.03 | 0.73 |
| **15** | 0.81 | 0.03 | 0.75 |
| **16** | 0.75 | 0.03 | 0.78 |
| **17** | 0.70 | 0.02 | 0.80 |
| **18** | 0.67 | 0.02 | 0.83 |
| **19** | 0.66 | 0.02 | 0.85 |
| **20** | 0.58 | 0.02 | 0.87 |
| **21** | 0.55 | 0.02 | 0.89 |
| **22** | 0.51 | 0.02 | 0.91 |
| **23** | 0.50 | 0.02 | 0.92 |
| **24** | 0.49 | 0.02 | 0.94 |
| **25** | 0.45 | 0.02 | 0.96 |
| **26** | 0.36 | 0.01 | 0.97 |
| **27** | 0.32 | 0.01 | 0.98 |
| **28** | 0.29 | 0.01 | 0.99 |
| **29** | 0.28 | 0.01 | 1.00 |

**Note:** There were 29 baseline baseline characteristics used in the PCA. These included: Age at index date, Male, White Race, Modified Charlson comorbidity score, history of coronary artery disease (with angina), history of heart failure, history of ischemic stroke, history of stroke or transient ischemic attack, history of myocardial infarction, history of diabetes mellitus, history of a major malignancy, history of malignancy (excluding skin cancer), history of coronary revascularization, history of depression, history of fractures, history of femur fracture, history of hip fracture, had a late referral to a nephrologist prior to starting renal replacement therapy, first modality used was in-center hemodialysis, first vascular access was a central venous catheter, last vascular access used prior to the index date was a central venous catheter, younger than 65 and at least one prescription in ODB in the 6 months prior to index date, had at least one prescription of calcium channel blocker in the six months prior to index date, had at least one diuretics prescription in the 6 months prior to index date, number of days spent in the hospital in the 12 months prior to index date, number of hospitalization visits in the 12 months prior to index date, number of general practitioner visits in the 12 months prior to index date, number of cardiology visits in the 12 months prior to index date, number of emergency department visits in the 12 months prior to index date.

** See definition of Eigenvalue at:^8^ <http://mathworld.wolfram.com/Eigenvalue.html>

PC= Principal component; We used the first ten principal components in the analysis, which explained 61% of the variation in the baseline data.

**Appendix 7**: We used the principal axis method to extract the principal components. A varimax (orthogonal) rotation followed the principal axis method. Only the first ten components displayed eigenvalues greater than 1 (see **Appendix 6)**, and the results of a scree test also suggested that only the first ten components were meaningful. Therefore, we retained the first ten components for rotation.

| **Baseline characteristic** | **Factor 1** | **Factor 2** | **Factor 3** | **Factor 4** | **Factor 5** | **Factor 6** | **Factor 7** | **Factor 8** | **Factor 9** | **Factor 10** |
| --- | --- | --- | --- | --- | --- | --- | --- | --- | --- | --- |
| **History of coronary artery disease (with angina)** | 7 | 69* | 1 | -4 | -7 | 0 | 0 | -1 | -6 | 11 |
| **Had at least one prescription of calcium channel blocker in the six months before the index date** | 0 | -8 | 0 | 5 | -1 | 1 | 0 | 72* | 5 | -10 |
| **History of congestive heart failure** | 16 | 53* | 4 | -12 | 12 | 3 | 3 | 16 | 12 | -5 |
| **Malignancy (excluding skin cancer)** | 4 | 1 | 0 | -12 | -2 | 1 | 84* | -2 | -1 | 12 |
| **Number of cardiology visits in the 12 months prior to index date** | 66* | 31 | -3 | 6 | -2 | -9 | -2 | -5 | -6 | -2 |
| **Modified Charlson comorbidity score** | 28 | 58* | 7 | -1 | 9 | 29 | 22 | 22 | 13 | -23 |
| **History of coronary revascularization** | 1 | 66* | -1 | 7 | -4 | -6 | -2 | -10 | -12 | 1 |
| **History of diabetes mellitus** | 9 | 39 | 3 | -3 | 2 | 20 | 3 | 39 | 7 | -41* |
| **Late referral to a nephrologist** | -2 | -2 | -2 | 14 | 57* | -3 | 4 | -20 | 14 | -4 |
| **Depression** | 14 | 10 | 11 | 16 | -6 | 18 | 4 | 26 | 24 | 49* |
| **Use of diuretics in the 6 months prior to index date** | -1 | 10 | -2 | -10 | 1 | -5 | -3 | 69* | -4 | 12 |
| **Number of emergency department visits in the 12 months prior to index date** | 69* | 5 | 1 | 19 | 3 | 4 | 3 | 13 | 4 | 14 |
| **First modality used was in-center hemodialysis** | 3 | -2 | 4 | -13 | 70* | 1 | -5 | 18 | -32 | 6 |
| **History of fractures** | 7 | 1 | 84* | -4 | 1 | 2 | 3 | 2 | 4 | 6 |
| **Number of general practitioner visits in the 12 months before the index date** | 54* | 0 | 7 | -18 | 4 | 13 | 4 | 0 | 7 | 3 |
| **Number of days spent in the hospital in the 12 months prior to index date** | 71* | -1 | 7 | -12 | 0 | 2 | -3 | -10 | 4 | 0 |
| **Number of hospitalization visits in the 12 months prior to index date** | 83* | 13 | 2 | 11 | 2 | 3 | 6 | 6 | 5 | 5 |
| **History of ischemic stroke** | 7 | 1 | -2 | 1 | -2 | 83* | -3 | -3 | 1 | -1 |
| **History of myocardial infarction** | 4 | 73* | 1 | -12 | 5 | 5 | -6 | -3 | -3 | 9 |
| **History of a major malignancy** | 3 | 0 | 0 | -2 | 4 | -3 | 87* | -1 | -1 | -3 |
| **Male** | 0 | 16 | -7 | 6 | 9 | 1 | 5 | -7 | -66* | 0 |
| **White Race** | 2 | 7 | 4 | -15 | 6 | 1 | 8 | -6 | -5 | 78* |
| **Patients was younger than 65 and at least one prescription in ODB in the 6 months prior to index date** | 0 | -5 | -2 | 87* | 1 | -2 | -4 | 9 | -3 | 1 |
| **Age at index date** | -2 | 14 | 7 | -84* | -2 | 6 | 11 | 18 | 9 | 10 |
| **History of femur fracture** | 3 | 3 | 78* | 1 | 1 | 0 | -3 | -2 | 2 | -2 |
| **History of hip fracture** | 3 | 4 | 89* | -5 | 1 | 1 | 1 | -1 | 3 | 5 |
| **First vascular access was a central venous catheter** | 6 | 8 | 2 | -2 | 82* | 3 | 2 | 6 | 16 | 1 |
| **The last access used before the index date was a central venous catheter** | 13 | 11 | 1 | -7 | 28 | 2 | 3 | -5 | 70* | 3 |
| **History of stroke or transient ischemic attack** | 4 | 8 | 3 | -8 | 3 | 83* | 0 | 1 | -1 | 7 |

**Note**: Values above were multiplied by 100 and rounded to the nearest integer. Values greater than the absolute value of 40 were flagged by an '*.'

**Appendix 8**: Hardware specification and optimization for running the constrained randomization process.

It took approximately 1 second to evaluate each randomization scheme's balance or a total of 83 hours (of CPU time) to assess all 300,000 allocation schemes (see **below** for hardware specification and optimization). From the 300,000 allocations, we constrained the randomization space to the 30,000 best allocations (i.e., 10% of the randomization space) and randomly selected 1000 allocations. All 1000 sampled allocations schemes were balanced on all constrained baseline characteristics in the Population for Randomization, regardless of the constraining method.

**Hardware**: We used a Windows 10 Intel(R) Core(TM) i7-7500U CPU @ 2.70GHz, 2904 Mhz with 2 Cores and 4 Logical Processors. This hardware had 12GB RAM.

**Optimization**: Rather than running the 300,000 allocation schemes sequentially, we parallelized the process by utilizing the "RSUBMIT" statement in SAS. Parallel processes allowed us to execute three statements in a remote SAS session with three logical processors. This method reduced our computation time by approximately a third.

**Appendix 9**: The percentage of times baseline characteristics were balanced across 1000 randomization schemes for the three techniques.

| **Baseline characteristic** | **Value** | **Constrained randomization method** | | |
| --- | --- | --- | --- | --- |
|  |  | **Unrestricted / Simple** | **Prognostic baseline characteristics** | **Principal components** |
| Neighbourhood Income Quintile | 1 | 77.5% | 77.0% | 75.3% |
|  | 2 | 98.8% | 98.5% | 98.8% |
|  | 3 | 98.7% | 98.7% | 99.0% |
|  | 4 | 91.9% | 95.6% | 93.6% |
|  | 5 | 89.0% | 91.9% | 92.8% |
|  | Missing | 100.0% | 100.0% | 100.0% |
| Patients < 65 years in ODB in the 6 months prior to index date | Yes | 99.8% | 99.7% | 99.9% |
| Alpha-Blockers | Yes | 88.6% | 92.0% | 92.9% |
| Benzodiazepine | Yes | 98.3% | 98.6% | 99.6% |
| Centrally Acting Antiadrenergic | Yes | 96.0% | 94.9% | 95.1% |
| Midodrine | Yes | 71.6% | 73.9% | 71.6% |
| Vasodilators | Yes | 96.6% | 98.1% | 97.4% |
| Anti-psychotics | Yes | 100.0% | 100.0% | 100.0% |
| Anti-depressants | Yes | 96.0% | 98.7% | 99.4% |
| Number of hypertensive subclasses prescribed in the six months before index date | Mean ± Standard deviation | 93.3% | 98.9% | 97.7% |
| Abdominal aortic aneurysm repair/aortic bypass | Yes | 100.0% | 100.0% | 100.0% |
| Atrial Fibrillation/Flutter | Yes | 99.7% | 100.0% | 100.0% |
| Acute Kidney Injury in the six months before the index date | Yes | 97.0% | 98.8% | 99.1% |
| Alcoholism | Yes | 100.0% | 100.0% | 100.0% |
| Arrhythmia | Yes | 99.9% | 100.0% | 100.0% |
| Coronary Artery Disease (with angina) | Yes | 96.3% | 98.7% | 96.1% |
| Dementia | Yes | 98.9% | 99.7% | 99.7% |
| Depression | Yes | 97.4% | 99.1% | 100.0% |
| Having any type of Fracture | Yes | 100.0% | 100.0% | 100.0% |
| Fracture of the Humerus | Yes | 100.0% | 100.0% | 100.0% |
| Fracture of the Pelvis | Yes | 100.0% | 100.0% | 100.0% |
| Fracture of the Femur | Yes | 100.0% | 100.0% | 100.0% |
| Fracture of the Hip | Yes | 100.0% | 100.0% | 100.0% |
| Fracture of the wrist | Yes | 100.0% | 100.0% | 100.0% |
| Hypertension | Yes | 100.0% | 100.0% | 100.0% |
| Hypotension | Yes | 100.0% | 100.0% | 99.9% |
| Subarachnoid Hemorrhage | Yes | 100.0% | 100.0% | 100.0% |
| Liver disease | Yes | 99.8% | 99.6% | 98.9% |
| Malignancy (excluding skin cancer) | Yes | 95.1% | 94.5% | 98.5% |
| Other Serious Illness that could shorten life expectancy to less than five years | Yes | 55.2% | 56.7% | 59.5% |
| Smoker | Yes | 96.8% | 99.5% | 100.0% |
| Syncope | Yes | 100.0% | 100.0% | 100.0% |
| Venous thromboembolism | Yes | 99.9% | 99.8% | 99.9% |
| Stroke/Transient ischemic attack (TIA) | Yes | 100.0% | 100.0% | 100.0% |
| Body mass index | Mean ± Standard deviation | 93.4% | 98.5% | 99.6% |
|  | Underweight | 100.0% | 100.0% | 100.0% |
|  | Normal | 99.2% | 99.5% | 99.9% |
|  | Overweight | 100.0% | 100.0% | 100.0% |
|  | Obese I | 100.0% | 100.0% | 100.0% |
|  | Obese II | 100.0% | 100.0% | 100.0% |
|  | Obese III | 99.4% | 99.9% | 100.0% |
|  | Missing | 96.2% | 98.7% | 96.3% |
| Abdominal/Renal ultrasound | Yes | 100.0% | 100.0% | 100.0% |
| Chest x-ray | Yes | 100.0% | 99.9% | 100.0% |
| Coronary angiogram | Yes | 96.7% | 97.1% | 96.6% |
| Coronary revascularization | Yes | 100.0% | 100.0% | 100.0% |
| Echocardiography | Yes | 98.5% | 99.6% | 99.7% |
| Holter monitoring | Yes | 99.2% | 99.9% | 99.5% |
| Stress test | Yes | 94.9% | 96.4% | 95.5% |
| Carotid endarterectomy | Yes | 100.0% | 100.0% | 100.0% |
| Number of cardiology visits in the year prior to the index date | Mean ± Standard deviation | 81.1% | 81.4% | 86.9% |
|  | 0 | 78.4% | 76.4% | 81.1% |
|  | 1 to 3 | 99.5% | 99.6% | 99.9% |
|  | 4 to 6 | 99.4% | 99.6% | 99.5% |
|  | 7 to 9 | 100.0% | 99.9% | 100.0% |
|  | 10+ | 92.1% | 92.0% | 94.6% |
| Number of general practitioner visits in the year before the index date | Mean ± Standard deviation | 59.9% | 55.6% | 57.4% |
|  | 0 | 78.4% | 76.4% | 81.1% |
|  | 1 to 3 | 99.5% | 99.6% | 99.9% |
|  | 4 to 6 | 99.4% | 99.6% | 99.5% |
|  | 7 to 9 | 100.0% | 99.9% | 100.0% |
|  | 10+ | 92.1% | 92.0% | 94.6% |
| Number of nephrology consults in the year prior to the index date | Mean ± Standard deviation | 77.4% | 95.5% | 86.9% |
|  | 0 | 78.4% | 76.4% | 81.1% |
|  | 1 to 3 | 99.5% | 99.6% | 99.9% |
|  | 4 to 6 | 99.4% | 99.6% | 99.5% |
|  | 7 to 9 | 100.0% | 99.9% | 100.0% |
|  | 10+ | 92.1% | 92.0% | 94.6% |
| Number of days spent in the hospital in the year prior to the index date | Mean ± Standard deviation | 92.2% | 98.1% | 98.7% |
|  | 0 | 78.4% | 76.4% | 81.1% |
|  | 1 to 3 | 99.5% | 99.6% | 99.9% |
|  | 4 to 6 | 99.4% | 99.6% | 99.5% |
|  | 7 to 9 | 100.0% | 99.9% | 100.0% |
|  | 10+ | 92.1% | 92.0% | 94.6% |
| Number of emergency department visits in the year before the index date | Mean ± Standard deviation | 90.8% | 97.1% | 97.7% |
|  | 0 | 78.4% | 76.4% | 81.1% |
|  | 1 to 3 | 99.5% | 99.6% | 99.9% |
|  | 4 to 6 | 99.4% | 99.6% | 99.5% |
|  | 7 to 9 | 100.0% | 99.9% | 100.0% |
|  | 10+ | 92.1% | 92.0% | 94.6% |
| Number of days spent in long-term care in the year prior to the index date | Mean ± Standard deviation | 99.3% | 100.0% | 99.9% |
| Number of dialysis sessions in the year prior to the index date | Mean ± Standard deviation | 84.2% | 83.4% | 83.9% |
| Height (cm) prior to starting dialysis | Mean ± Standard deviation | 100.0% | 100.0% | 100.0% |
| Weight (kg) prior to starting dialysis | Mean ± Standard deviation | 99.1% | 99.9% | 99.9% |
| Urea test result before starting dialysis | Mean ± Standard deviation | 99.5% | 99.8% | 100.0% |
| Hemoglobin test results prior to starting dialysis | Mean ± Standard deviation | 85.1% | 87.7% | 88.6% |
| Creatinine test result prior to starting dialysis | Mean ± Standard deviation | 77.8% | 88.5% | 97.1% |
| eGFR using CKD EPI | Mean ± Standard deviation | 83.9% | 89.1% | 96.6% |
| Serum albumin test result before starting dialysis | Mean ± Standard deviation | 67.5% | 69.3% | 72.8% |

++ The covariate constrained randomization also included two cluster-level baseline characteristics: cluster size at the time of randomization and the historical rate of cardiovascular-related death and hospitalization for myocardial infarction, ischemic stroke, and heart failure.

# **References**

1. UCLA: Statistical Consulting Group. Factor analysis: SAS annotated output. Accessed February 21, 2020. https://stats.idre.ucla.edu/sas/output/factor-analysis/

2. Hatcher L, O’Rourke N. *A Step-by-Step Approach to Using SAS® for Factor Analysis and Structural Equation Modeling*. SAS Institute; 2013.

3. Quinn RR, Laupacis A, Austin PPC, et al. Using administrative datasets to study outcomes in dialysis patients: a validation study. *Med Care*. 2010;48(8):745-750. doi:10.1097/MLR.0b013e3181e419fd

4. Kokotailo R a, Hill MD. Coding of stroke and stroke risk factors using international classification of diseases, revisions 9 and 10. *Stroke*. 2005;36(8):1776-1781. doi:10.1161/01.STR.0000174293.17959.a1

5. Andrade SE, Harrold LR, Tjia J, et al. A systematic review of validated methods for identifying cerebrovascular accident or transient ischemic attack using administrative data. *Pharmacoepidemiol Drug Saf*. 2012;21 Suppl 1:100-128. doi:10.1002/pds.2312

6. Juurlink D, Preyra C, Croxford R, et al. *Canadian Institute for Health Information Discharge Abstract Database : A Validation Study*.; 2006.

7. Schultz SE, Rothwell DM, Chen ; Z, Tu ; K, Chen Z, Tu K. Identifying cases of congestive heart failure from administrative data: a validation study using primary care patient records. *Chronic Dis Inj Can*. 2013;33(3):160-166.

8. Weisstein EW. Eigenvalue. Accessed November 20, 2019. http://mathworld.wolfram.com/Eigenvalue.html
